# Preclinical data on the combination of cisplatin and anti-CD70 therapy in non-small cell lung cancer as an excellent match in the era of combination therapy

**DOI:** 10.18632/oncotarget.18202

**Published:** 2017-05-23

**Authors:** Julie Jacobs, Vanessa Deschoolmeester, Christian Rolfo, Karen Zwaenepoel, Jolien Van den Bossche, Christophe Deben, Karen Silence, Hans de Haard, Christophe Hermans, Sylvie Rottey, Christel Vangestel, Filip Lardon, Evelien Smits, Patrick Pauwels

**Affiliations:** ^1^ Center for Oncological Research, University of Antwerp, Antwerp, 2610 Wilrijk, Belgium; ^2^ Department of Pathology, Antwerp University Hospital, Antwerp, 2650 Edegem, Belgium; ^3^ Department of Oncology, Antwerp University Hospital, Antwerp, 2650, Edegem, Belgium; ^4^ Phase 1-Early Clinical Trials Unit, Antwerp University Hospital, Antwerp, 2650 Edegem, Belgium; ^5^ Argenx BVBA, Ghent, 9052 Zwijnaarde, Belgium; ^6^ Department of Medical Oncology, Ghent University Hospital, 9000 Ghent, Belgium; ^7^ Molecular Imaging Center Antwerp (MICA), University of Antwerp, Antwerp, 2610 Wilrijk, Belgium; ^8^ Laboratory of Experimental Hematology (LEH), Vaccine and Infectious Disease Institute, University of Antwerp, Antwerp, 2610 Wilrijk, Belgium

**Keywords:** non-small cell lung cancer, combination therapy, natural killer cell, CD70, chemotherapy

## Abstract

In contrast to the negligible expression of the immunomodulating protein CD70 in normal tissue, we have demonstrated constitutive overexpression of CD70 on tumor cells in a subset of primary non-small cell lung cancer (NSCLC) biopsies. This can be exploited by CD70-targeting antibody-dependent cellular cytotoxicity (ADCC)-inducing antibodies. Early clinical trials of these antibodies have already shown promising results in CD70-positive malignancies.

In this study, we explored the potential of cisplatin to induce CD70 expression in NSCLC. Using real-time measurement tools, we also assessed the efficacy of a combination regimen with cisplatin and anti-CD70 therapy under normoxia and hypoxia. We identified an induction of CD70 expression on lung cancer cells upon low doses of cisplatin, independent of oxygen levels. More importantly, the use of cisplatin resulted in an enhanced ADCC-effect of anti-CD70 therapy. As such, this combination regimen led to a significant decrease in lung cancer cell survival cell survival, broadening the applicability the applicability of CD70-targeting therapy.

This is the first study that proves the potential of a combination therapy with cisplatin and CD70-targeting drugs in NSCLC. Based on our data, we postulate that this combination strategy is an interesting approach to increase tumor-specific cytotoxicity and reduce drug-related side effects.

## INTRODUCTION

Non-small cell lung cancer (NSCLC) retains its position as the most lethal type of cancer worldwide with around 1.3 million deaths each year and a marginally improving 5-year overall survival rate which remains below 20% [1]. These data point towards the continued need for new therapeutic modalities. Recent insights into the biology of the immune response have led to a wave of clinical trials involving immunotherapy for lung cancer. Nonetheless, the immune suppressive and heterogeneous nature of this tumor generates variable success rates of immunotherapy in NSCLC patients, leaving room leaving room for improvement.

The expression of CD70, a member of the tumor necrosis factor family (TNF), is normally restricted to activated T and B cells and mature dendritic cells [2]. In contrast, we have previously revealed constitutive overexpression of CD70 on malignant cells in 16% of NSCLC tumor specimens [3]. Since CD70 is absent on normal epithelial tissue, the overexpression on tumor cells can be safely exploited by CD70-targeting antibody-dependent cellular cytotoxicity (ADCC)-inducing antibodies, such as ARGX-110 [4]. Furthermore, it has been described that tumor-specific upregulation of CD70, through its unique receptor CD27, can facilitate immune evasion by increasing the proliferation of suppressive regulatory T cells, inducing T cell apoptosis and skewing T cells towards a T cell exhaustion profile[5–8]. Although we believe that anti-CD70 therapy holds great potential as monotherapy, this strategy would only be applicable to patients with CD70-positive tumors. Recently, the immunomodulatory properties of chemotherapy in combination with immunotherapy have been demonstrated [9]. Although chemotherapy has long been considered immune suppressive, recent studies have shown that in addition to its direct cytotoxic effects on cancer cells, certain chemotherapeutics can elicit changes in the tumor microenvironment that render cells more sensitive to an efficient immune cell attack [10]. In the present study, we evaluated a new combinatorial strategy with cisplatin (CDDP), first-line treatment in NSCLC, to maximize the efficacy of anti-CD70 therapy. We examined the induction of CD70 expression upon CDDP treatment *in vitro* and *in vivo.* We further evaluated the ADCC effect and immune stimulatory potential of anti-CD70 therapy (aCD70) upon sequential treatment with low-doses of CDDP. Finally, we assessed the therapeutic efficacy of the combination regimen under hypoxic conditions as regions within the tumor with different oxygen levels often characterize therapy resistance.

## RESULTS

### Dose-response analysis of CDDP in NSCLC cell lines

**Figure 1:**
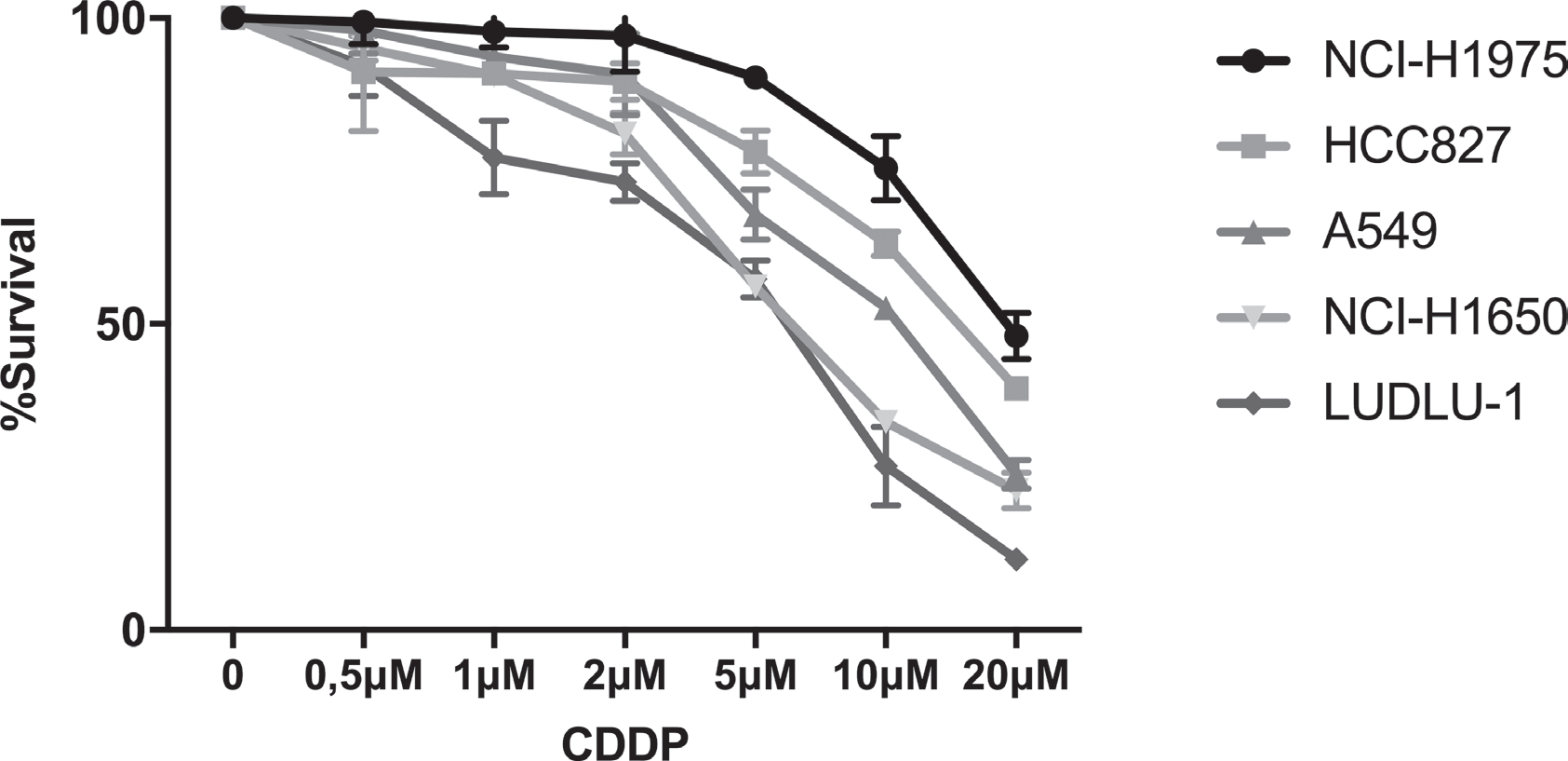
Dose-response curve of CDDP in NSCLC cell lines Survival curve after 24 h of treatment with CDDP (0–20 mM) in the NCI-H1975, HCC827, A549, NCI-H1650 and LUDLU-1 cells.

The cytotoxicity of CDDP monotherapy was assessed in a panel of five NSCLC cell lines, varying in genetic aberrations and histological subtype (Supplementary Table 1). Cells were treated with CDDP (0–20 µM) for 24 h and chemosensitivity was assessed by the Sulforhodamine-B assay. LUDLU-1 (IC_50_: 5.46µM ± 0.93 µM) was most sensitive to treatment, followed by NCI-H1650 (IC_50_: 6.51 µM ± 0.46 µM). NCI-H1975 (IC_50_: 19.34µM ± 1.72 µM) and HCC827 (IC_50_: 15.95 µM ± 1.37 µM) cells were most resistant to CDDP treatment (Figure 1). Because dose-response analysis of A549 cells (IC_50_: 10.04 µM ± 0.72 µM) was in-between our panel of NSCLC cell lines, we considered the doses equivalent to 20% (3.5 µM), 40% (7 µM) and 60% (13 µM) of CDDP-induced growth inhibition in the A549 cells as respectively low, medium and high doses.

### CDDP induces/increases CD70 expression on protein and mRNA level

**Figure 2:**
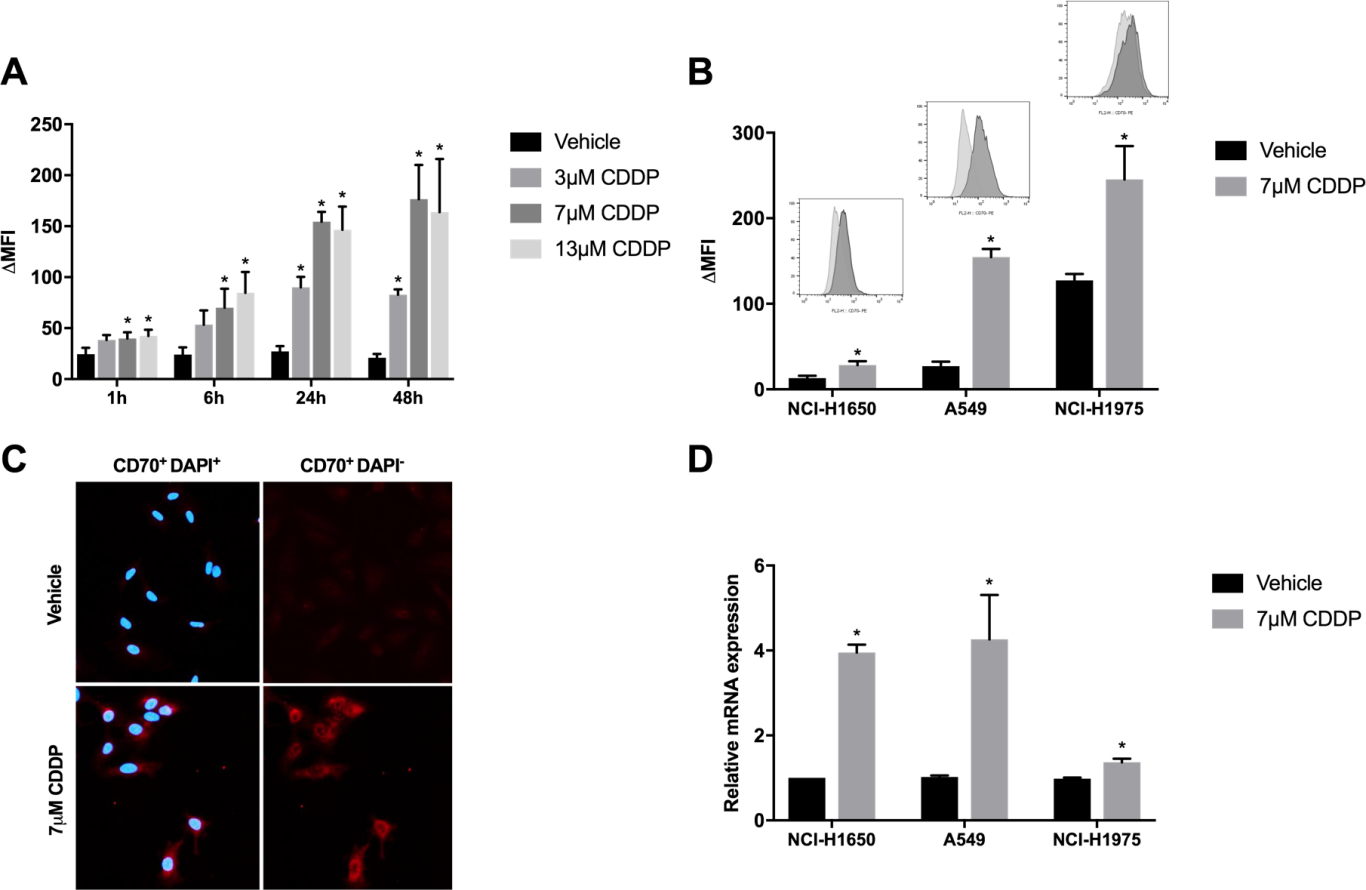
Changes in CD70 protein- and mRNA expression levels in response to CDDP-therapy (**A**) A549 cells were treated with vehicle or CDDP (3.5 µM, 7 µM or 13 µM) for 24 h. CD70 expression levels were determined 1 h, 6 h, 24 h and 48 h after treatment. Graph represents ∆ mean fluorescence intensity (∆MFI) of three independent experiments; (**B**) NCI-H1975, A549 and NCI-H1650 cells were treated with vehicle or CDDP (7 µM) for 24 h. Graph represents ∆MFI of three independent experiments. In addition, representative histogram plots of vehicle- and CDDP-treated cells are displayed; (**C**) CD70 expression was measured by immunofluorescence (red) after treatment with vehicle or CDDP (7 µM) for 24 h in the A549 cells. Nuclei were stained by DAPI (blue); (**D**) CD70 mRNA levels were determined 24 h after treatment with vehicle or CDDP (7 µM). Graph represents relative mRNA levels in comparison to vehicle-treated cells. *P < 0.05, significant increase compared to vehicle.

To analyze the impact of CDDP treatment on CD70 expression, 3 NSCLC cell lines (NCI-H1650, A549, NCI-H1975) were selected out of the panel of NSCLC cell lines, based on their aberrations in CD70 expression levels (CD70^-^, CD70^+^ and CD70^++^) by flow cytometry and screened for membranous CD70 expression levels in response to CDDP treatment. Cells were treated for 24 h with vehicle (0 µM), low (3.5 µM), medium (7 µM) or high (13 µM) doses of CDDP (Figure 1). Thereafter, CD70 protein levels were assessed at different time points (1 h, 6 h, 24 h, 48 h) in a propidium iodide (PI)-negative cell subset. As shown for the A549 cells, the highest induction of membranous CD70 was seen 24 h and 48 h after treatment with 7 µM of CDDP (Figure 2A). CD70 protein levels were also significantly upregulated in a very strong (NCI-H1975) and very weak (NCI-H1650) CD70^+^ expressing cell line (Figure 2B). In line with these results, immunofluorescence demonstrated a marked induction of CD70 protein levels after treatment with CDDP compared to vehicle (Figure 2C). To demonstrate that the observed effect was attributed to increased transcription of CD70, CD70 mRNA levels were screened after treatment with vehicle or CDDP. Twenty-four hours after treatment, an average 1.4-, 4.3- and 4.0- fold increase of CD70 mRNA could be detected in CDDP-treated NCI-H1975, A549 and NCI-H1650 cells, respectively (Figure 2D). To examine whether changes in DNA methylation contributed to CDDP-induced CD70 overexpression, methylation of 4 CG pairs located between 581 and 288 base pairs upstream of the TNFSF7 locus, was analyzed using bisulphite-converted genomic DNA in NCI-H1975 cells treated with vehicle or 7 µM CDDP (Supplementary Materials and Methods Supplementary Table 1). As shown in Supplementary Figure 1, the induction of CD70 protein levels could not be attributed to variations in the methylation status of the CD70 promotor region in NSCI-H1975 cells. In summary, these data indicate that CDPP treatment induces CD70 expression by increased levels of CD70 mRNA.

### Increased tumor-specific CD70 protein levels after induction chemotherapy

**Figure 3:**
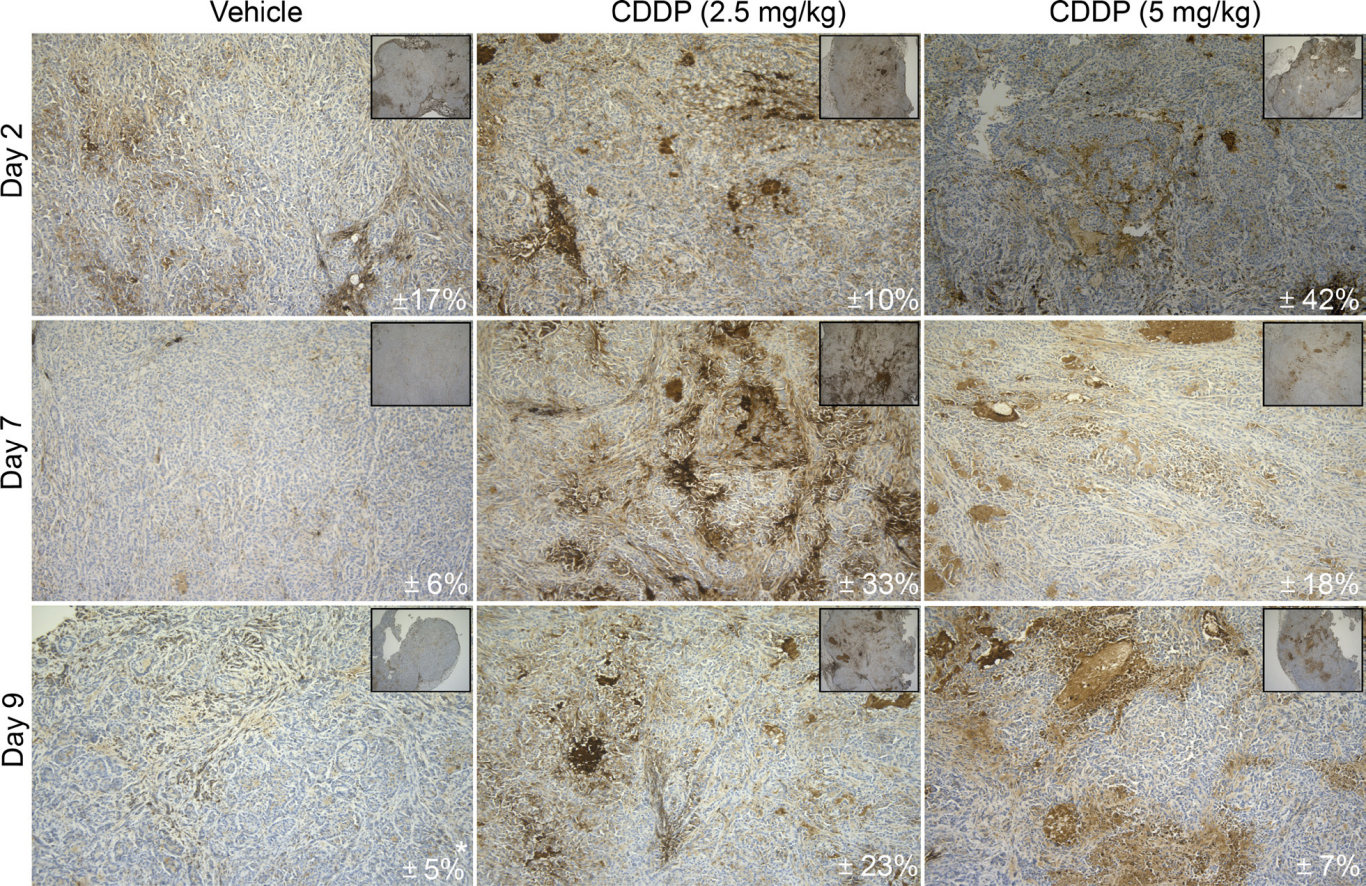
*In vivo* CD70 protein analysis upon CDDP-treatment CD-1 nude mice were inoculated with A549 cells in the right hind leg. Tumor-bearing mice were randomly divided into 3 groups and treated intraperitoneally with vehicle (0.9% NaCl), low (2.5 mg/kg) or high (5 mg/kg) doses of CDDP on day 0 and day 7. Mice were sacrificed at different time points (Day 2, Day 7, Day 9) to evaluate the expression of CD70 by IHC. Figure shows representative sections of CD70 expression levels in all conditions. Mean CD70 percentages (*N* = 3; *N = 2) are depicted in every right corner. Magnitude 10× and 100×.

Next, the CDDP-induced expression of CD70 was examined *in vivo* using A549 tumor-bearing CD1 nude mice to verify the expression upon CDDP-treatment and the effects of multiple treatment regimens. Therefore, tumor-bearing mice were treated with vehicle (0.9% NaCl), low (2.5 mg/kg) or high (5 mg/kg) doses of CDDP at day 0 and day 7. As shown in Figure 3, a high dose of CDDP (5 mg/kg) resulted in a peak in CD70 expression two days’ post-treatment. On the other hand, CD70 overexpression upon low doses of CDDP was only observed seven days’ post-treatment. Interestingly, at day 9 we could observe stable CD70 overexpression upon low dose of CDDP (2.5 mg/kg) whereas a decrease was seen in the high-dose group. These findings indicate that the post-therapy increase in CD70 expression was slightly delayed in the low treatment group but more stable over time.

### Combining CDDP with aCD70 significantly decreases tumor cell proliferation as opposed to single treatment regimens due to increased ADCC

**Figure 4:**
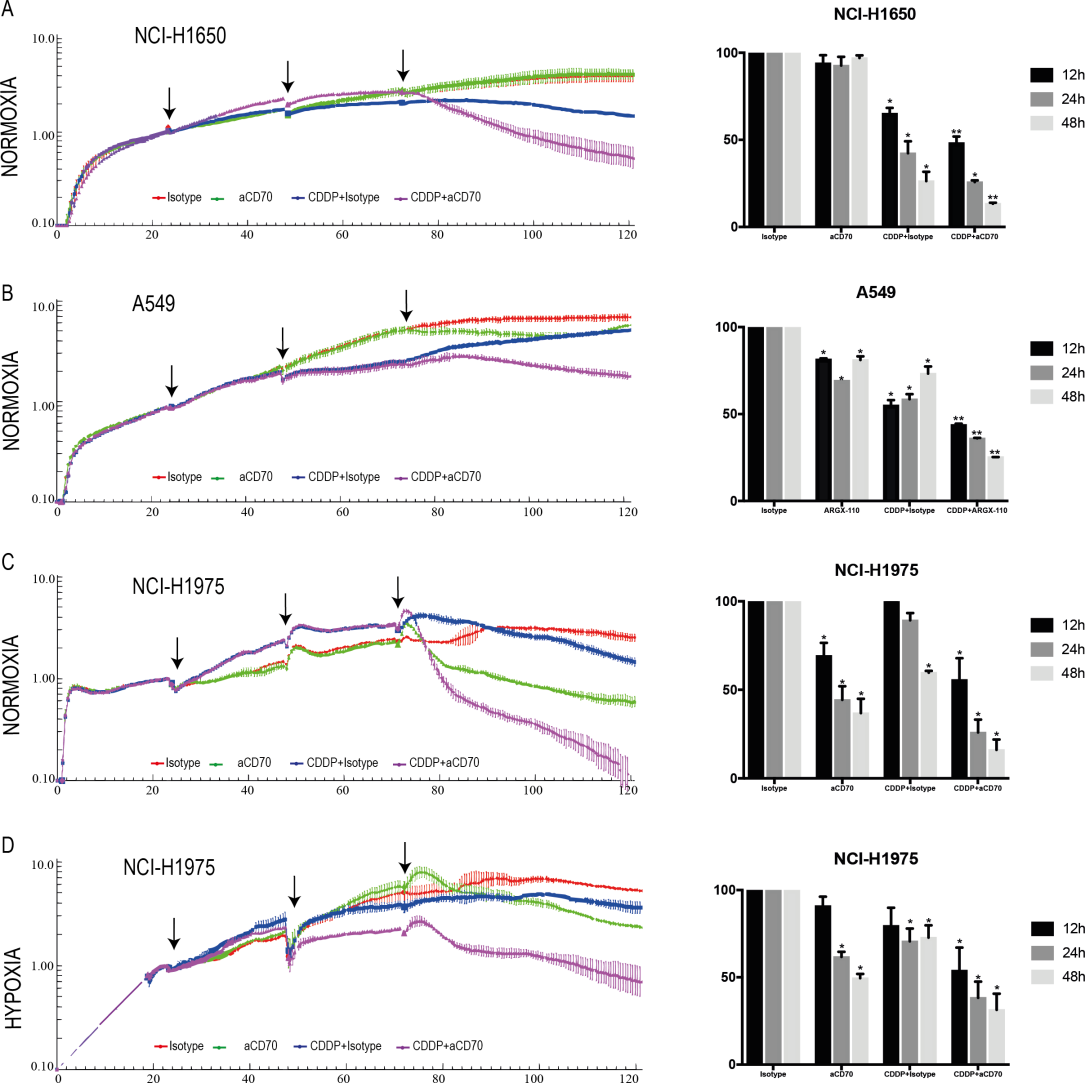
Combining CDDP with aCD70 significantly decreases tumor cell proliferation due to increased ADCC Left: NCI-H1650, A549, and NCI-H1975 cells were treated with vehicle or CDDP (24 h, 7 µM, 1st arrow). Cells were washed (2nd arrow) and aCD70 (0.5 µM) or isotype control (0.5 µM) was added to the medium in combination with NK cells (E/T=5/1) from healthy volunteers (3rd arrow). Cell survival was assessed up to 48 h after sequential treatment in 5 conditions: Vehicle, Isotype control (red), aCD70 (green), CDDP + isotype control (blue), CDDP + aCD70 (Purple). Left: Figure shows the well impedance of 1 representative donor, expressed by the cell index as a measure of viability, analyzed using the xCELLigence RTCA system. Cell indexes were normalized with the last point before compound addition. (**A-B-C**) Experiments were assessed under normoxic conditions. (**D**) Cells were seeded overnight under normoxic conditions. Thereafter experiments were assessed in an anaerobic chamber (O_2_: 0.1 – 1%). Lines represent the mean ± SEM. Right: Percentage of cell survival 12 h, 24 h and 48 h after sequential treatment. Bars represent the mean ± SEM. For all experiments, two replicates of the same condition were measured and run in parallel with NK cells from three different donors. *P < 0.05: significant decrease in cell survival compared to vehicle; **P < 0.05: significant decrease in cell survival compared to all other conditions.

We hypothesized that the significant accumulation of membranous CD70 upon CDDP-treatment could lead to an enhanced ADCC-effect of aCD70. To confirm this hypothesis, the ADCC potential of aCD70 in this combination regimen was evaluated in real-time using the xCELLigence RTCA system. Twenty-four hours after treatment of the cancer cells with medium doses of CDDP (24 h, 7 µM), natural killer (NK) cells (E/T=5/1) were cocultured with the cancer cells and treated with aCD70 (48 h, 0.5 µM) or isotype control (48 h, 0.5 µM). In the CD70^−^ cell line, NCI-H1650, the anti-proliferative effect of aCD70 in single treatment regimen was negligible as compared to isotype control (Figure 4A). Treatment with CDDP + isotype (CDDPi) on the other hand resulted in 26.34 ± 3.09% cell survival after 48 h as opposed to untreated cells. Interestingly, the combination of aCD70with medium doses of CDDP decreased proliferation significantly with an additional 12.99 ± 0.36% compared to CDDPi. In the weak CD70^+^ A549 cells, the ADCC-effect of aCD70 in monotherapy was more pronounced, resulting in 69.28 ± 0.02% and 81.09 ± 1.71% cell survival 24 h and 48 h post-treatment, respectively (Figure 4B). Yet again, the most significant decrease in cell survival was seen in the sequential combination regimen of CDDP and aCD70 (36.02 ± 0.25% (24 h) and 25.26 ± 0.04% (48 h) cell survival). In comparison to the other cell lines, the ADCC-effect of aCD70 in monotherapy was most pronounced in the strong CD70^+^ NCI-H1975 cells with only 36.87 ± 4.68% cell survival 48 h after treatment (Figure 4C). Even so, sequential administration of CDDP and aCD70 resulted in a clear additional decrease in cell survival of 20.72 ± 3.34% and 43.51 ± 3.34% compared to single treatment of aCD70 or CDDP, respectively. To confirm cell death, the combination strategy in the NCI-H1975 cell line was also examined by the evaluation of caspase 3/5, using the incuCyte^™^ as displayed in Supplementary Figure 2. Here, cell death was most pronounced in the combination therapy, supporting our previous results. In conclusion, we have revealed strong efficacy of a combination regimen with medium doses of CDDP and aCD70 in all cell lines tested.

### Strong efficacy of CDDP in combination with aCD70 under hypoxic conditions

**Figure 5:**
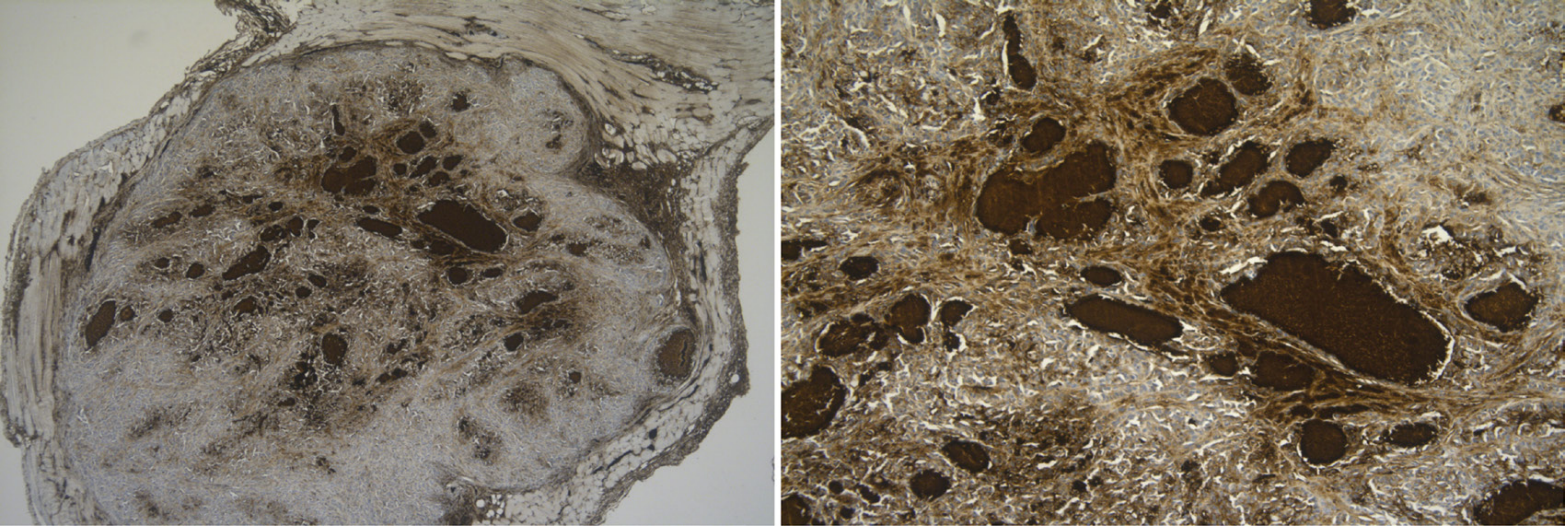
*In vivo* CD70 protein analysis in hypoxic regions Tumor-bearing CD1-nude mice were sacrificed to evaluate CD70 expression levels. Figure shows high CD70 expression levels in hypoxic regions surrounding necrotic areas. Magnitude 10× (left) and 100× (right).

Hypoxia is an important contributor to the heterogeneity of the tumor microenvironment in solid tumors, driving adaptations which are essential for the survival and metastatic capabilities of tumor cells [11]. Therefore, experiments were also conducted under hypoxic conditions. Interestingly, we noticed a higher CD70 baseline expression under hypoxic conditions in all cell lines tested (data not shown). Likewise, our immunohistochemistry (IHC) data on an *in vivo* mice model demonstrated a strong CD70 overexpression on hypoxic regions of the tumor, as shown in Figure 5.

Nonetheless the increased baseline CD70 expression under hypoxia, *in vitro* experiments demonstrated a similar induction of membranous CD70 levels 24 hours’ post-treatment with CDDP (24h, 7µM). In a final set of experiments, the ADCC potential of aCD70 after CDDP treatment was evaluated in the NCI-H1975 cell line using the xCELLigence RTCA system under reduced oxygen conditions (0.1–1% O_2_). In comparison to the treatment of the cells with CDDPi under normoxic conditions, the anti-proliferative effect was much less pronounced under hypoxia (Figure 4D). Forty-eight hours’ post-treatment, the greatest decrease in cell survival was seen after sequential treatment of CDDP and aCD70 with an additional decline of 41.44 ± 5.33% and 18.19 ± 5.33% compared to single treatment of aCD70 or CDDP, respectively. Hence, we can conclude that even under hypoxic conditions the use of CDDP can increase membrane CD70 expression levels and therefore enhance the ADCC-potential of aCD70.

## DISCUSSION

In this study, we are the first to demonstrate the induction of CD70 expression on the membrane of NSCLC cell lines upon CDDP treatment, a key drug for NSCLC treatment, even when administered at low doses. More importantly, the provision of CDDP treatment resulted in enhanced NK-cell mediated cellular cytotoxicity of ARGX-110, a CD70-specific mAb, in all cell lines tested.

Previous studies of our group have demonstrated overexpression of the tumor antigen, CD70, on malignant cells in 16% of NSCLC patients and its absence on normal lung tissue, making it an attractive target for antibody-based therapies [3]. Although our results demonstrated the potential of anti-CD70 therapy in NSCLC, this strategy would only be applicable to patients with CD70-positive tumors. However, here we have shown the increase of CD70 levels upon CDDP treatment, both on protein and mRNA level, thereby expanding the therapeutic window of anti-CD70 antibody treatment. Furthermore, we observed this overexpression in cells that survived CDDP treatment and therefore the induction of CD70 expression might also be linked with CDDP-resistance in NSCLC. In advanced ovarian cancer, Liu N et al. [12] described a similar phenomenon, with increased CD70 expression levels in patients resistant to CDDP-based adjuvant chemotherapies. However, the underlying mechanism of this overexpression was not investigated. In literature, it has been stated that the methylation status of CG pairs, located between 581 and 288 base pairs upstream of the TNFSF7 locus can influence CD70 transcription [13]. Because CDDP treatment can influence epigenetics, we have investigated these CG pairs upon CDDP treatment, but we did not notice any deviations in methylation status. As such, it is very likely that CDDP is affecting the regulation of transcription factors that bind to the CD70 promotor region such as AP-1 [14, 15]. Of note, CD70 upregulation was observed in cell lines with various p53 status, pointing towards a p53-independent mode of action. This is in line with previous experiments by Wisschusen et al. who demonstrated that radioinducibility of the CD70 gene did not require wild-type p53 activity and that this was not a response to genotoxic stress [16]. According to this study, the induction of CD70 upon irradiation was driving immune escape by the induction of apoptosis in peripheral blood mononuclear cells (PBMCs). The observed CDDP-mediated CD70 expression might also pave the way towards immune escape as the CD70/CD27 axis has been linked with increased regulatory T cell counts in NSCLC specimens [3]. Therefore, our findings suggest that CD70-targeted therapies might be an interesting treatment choice to combine with CDDP to release the brakes on the immune system. In addition, it was demonstrated that some chemotherapeutic agents feature the ability to directly activate immune effectors such as NK cells [17]. In a clinical setting, such a synergistic combination therapy has already been proven effective with paclitaxel and trastuzumab [18]. Interestingly, CDDP treatment has previously shown to upregulate important ligands for NK cell-mediated eradication of tumor cells, including androgen receptor-UL16-binding protein 2 (ULBP2) and MHC class I chain-related molecule A and B (MICA A/B) in hepatocellular carcinoma and NSCLC, respectively [19, 20]. NK cells also play a pivotal role in mediating ADCC, whereby binding of their FcγRIII receptors to Fc portions of antibody-coated target cells stimulates the release of cytotoxic molecules to kill the target cells. As such, we hypothesized that due to the favorable effects on NK cells and the increase in CD70 expression upon CDDP treatment, the sequential administration of CDDP and aCD70 could increase antibody-coverage and improve NK-mediated lysis of the tumor cells [4]. In this study, we revealed an enhanced efficacy of aCD70 upon treatment with low doses of CDDP by an increase in ADCC leading to tumor cell apoptosis. Moreover, by the induction of *de novo* CD70 expression upon CDDP treatment, significant reduction in cell survival was also seen in cells that did not show any response to aCD70 in monotherapy.

Hypoxia readily occurs in the majority of solid tumors, including NSCLC, and has been proven to contribute to tumor progression, metastasis and therapy resistance [21]. In addition, the natural killing capability of NK cells is compromised in a hypoxic microenvironment mediated through downregulation of important activating receptors [22]. Nevertheless, ADDC activity of NK cells is preserved under hypoxia [22]. Hence, our combination strategy of aCD70 and CDDP treatment was also explored under reduced oxygen levels. Firstly, a higher baseline CD70 expression was observed *in vitro* and *in vivo* under reduced oxygen levels in all cell lines. Likewise, it was recently described that CD70 expression is driven by hypoxia-inducible factor a in clear renal cell carcinoma [23]. We also found a strong induction of membranous CD70 expression upon CDDP treatment, which was similar to the effect of CDDP under normoxic condition. Finally, we could trigger efficient NK-cell mediated killing following treatment of CDDP and aCD70 indicating that hypoxia is not a limiting factor for this promising combination strategy.

## MATERIALS AND METHODS

### Cell lines and cell culture

The human NSCLC cell lines NCI-H1975, NCI-H1650, HCC827, LUDLU-1 and A549 were purchased from the American type cell culture collection (ATCC, Rockville MD, USA). The A549 cell line was cultured in DMEM supplemented with 10% fetal bovine serum (FBS), 1% penicillin/streptomycin and 1% L-glutamine (Life Technologies, Merelbeke, Belgium). NCI-H1975, NCI-H1650, HCC827 and LUDLU-1 cells were cultured in RPMI supplemented as described above with addition of 1mM sodium pyruvate (Life Technologies). Cells were grown as monolayers and were maintained in exponential growth at 5% CO_2_/95% air in a humidified incubator at 37°C to obtain normoxic conditions and in a humidified Bactron IV anaerobic chamber (Shel Lab, OR, USA, 1% O_2_, 5% CO_2_, 95% N_2_) to obtain hypoxic conditions. Cells used for experiments in hypoxic conditions were first grown overnight under normoxia to allow the cells to attach to the bottom. Cell cultures were regularly tested for absence of mycoplasma using the Mycoalert^®^ Mycoplasma detection kit (Lonza, Verviers, Belgium) .

### Purification of human natural killer (NK) cells

Human peripheral blood mononuclear cells (PBMC) were isolated by Ficoll-Paque Plus gradient separation (Amersham Biosciences, Uppsala, Sweden) from buffy coat preparations of healthy donors, provided by the Blood Transfusion Centre of Red Cross-Flanders (Mechelen, Belgium). Primary NK cells were negatively isolated from PBMC using the NK cell isolation kit (Miltenyi Biotec, Utrecht, The Netherlands; CD56^+^CD3^-^ cells, purity 88.1 ± 4.7%) as previously described. [3] In all experiments, NK cells of 3 different healthy donors were used.

### Cytotoxicity assays

*SRB assay*. To evaluate cytotoxicity of CDDP, cells were seeded in 96-well plates, incubated overnight and treated for 24 h with CDDP (0–20 µM) as single agent. Forty-eight hours after treatment, cell monolayers were fixed with 10% trichloroacetic acid for 1 h at 4°C and stained with 100 μl 0.1% sulforhodamine B, as previously described [24]. The IC_20_, IC_40_ and IC_60_ value, the concentration of the drug that leads to 20%, 40% and 60% growth inhibition respectively, was calculated using the WinNonlin software (Pharsight, CA, USA). All experiments were performed at least in triplicate.

*xCELLigence*. Real-time monitoring of cell viability was performed using the xCELLigence RTCA DP instrument (ACEA, Biosciences, San Diego, USA) as previously described. Shortly, target cells (T) were seeded at 3.5 × 10^3^ cells/well, incubated overnight and exposed to 7 µM CDDP (IC_20-_value of A549 cell line) or vehicle (phosphate buffered saline (PBS) for 24 h. One day after CDDP treatment, cells were treated with ARGX-110, further mentioned as aCD70 (argenx BVBA, Zwijnaarde, Belgium, 0.5 µM) or isotype control (I5029, Sigma-Aldrich, 0.5µM) in combination with effector (E) NK cells (E/T=5/1). Real-time measurements ended 6 days after initiation of the experiment. In each experiment, 2 replicates of the same condition were used and run in parallel with NK cells from 3 different donors.

### CD70 expression analysis

#### Flow cytometry

Cells were treated with 7 µM CDDP or vehicle for 24 h. Membrane CD70 expression was assessed by flow cytometry as previously described [3]. For experiments performed under hypoxic conditions, cells were fixed in 4% Formaldehyde (10 min) prior to primary antibody incubation. The signal for aspecific binding (anti-human IgG phycoerythrin (PE)-conjugated antibody, eBioscience, San Diego, USA) was subtracted from the measured fluorescence intensities (=∆MFI). Dead cells were excluded from analysis by staining with PI.

#### Immunofluorescence

Cells were treated with 7 µM CDDP or vehicle for 24 h. Cells were fixed in methanol, blocked with 1% BSA/PBS for 1h and incubated overnight with anti-CD70 (Clone 301731, 1/40, R&D systems, Abingdon, United Kingdom) at 4°C. Goat anti-mouse IgG secondary antibody, Alexa Fluor 555 conjugate (1/800, Thermo Fisher Scientific, Nepean, Canada) was used for 1 h as secondary antibody. Slides were counterstained with DAPI and mounted. Acquisition was performed by Evos Cell Imaging system (Thermo Fisher Scientific).

## IHC

Twenty-seven tissue specimens were fixed in 4% formaldehyde for 6–18 h and paraffin embedded on a routine basis. CD70 IHC (Clone 301731 diluted 1/40 for 20 min, R&D) was performed at room temperature on a DAKO autostainer Link 48 instrument using the Envision FLEX+ detection kit (DAKO) as previously described [3]. Scoring was performed by one pathologist, positive staining was assigned when tumor cells of any intensity and any CD70 distribution (membranous, cytoplasmic) showed specific CD70 staining.

### CD70 mRNA levels

*Real-time PCR (RT-PCR).* Cells were treated with 7 µM CDDP or vehicle for 24 h. RNA was isolated after 24 h of treatment using the TRIzol^®^ method (Life Technologies). Total RNA-yield and quality were measured using the NanoDrop^®^ ND-1000 (Thermo Fisher Scientific) and samples were stored at −80°C. RT-PCR was performed using the Power SYBR Green RNA-to-C_T_-Step kit (Applied Biosystems, Ghent, Belgium) on the LightCycler480 (Roche, Vilvoorde, Belgium) according to the manufacturer’s instructions with a total of 20 ng RNA. The optimal number and type of housekeeping genes (GAPD, RPLA13 and SDHA-1) were determined using the qbasePLUS software (Biogazelle, Zwijnaarde, Belgium) [24].

### *In vivo* A549 xenograft model

Female CD-1 athymic nude mice (*N* = 48, Charles River Laboratories, Calco, Italy) were purchased at an age of 6 weeks. Animals were group-housed in individually ventilated cages (3–8/cage) under specific pathogen free conditions with a 12 h day/night cycle and food and water ad libitum. A549 cells were harvested and resuspended in sterile PBS at a concentration of 4 × 10^7^ viable cells per ml. CD-1 nude mice were inoculated with 100µl of cell suspension in the right hind leg. Experiments were initiated when tumors reached a volume of approximately 100 mm³, ±4 weeks after inoculation. Tumor-bearing mice were randomly divided into 3 groups (*N* = 3) based on different treatment doses. CDDP was dissolved in PBS and applied intraperitoneally at low (2.5 mg/kg) or high (5 mg/kg) doses on day 0 and day 7. The vehicle group (0.9% NaCl) and drug treatment groups were housed separately. To evaluate the expression of CD70 during treatment, tumor-bearing mice were sacrificed at different time points (Day 2, Day 7, Day 9) and tumors were embedded in paraformaldehyde for the assessment of CD70 IHC, as described above. The experimental protocol was approved by the Ethical Committee for Animal Testing (N° 2016–29) and all applicable institutional and European guidelines for the care and use of animals were followed.

### Statistical analysis

Statistical significance for the *in vitro* experiments was determined by a one-way ANOVA test, followed by Tukey’s post hoc test whereby a *p*-value less than 0.05 was considered significant (two tailed). All analyses were conducted using SPSS version 23 (SPSS Inc., Chicago, IL, USA).

## CONCLUSIONS

In this study, we have identified a novel mechanism to treat patients with non-small cell lung cancer. The administration of CD70-targeted therapy with the first-line chemotherapeutic agent, Cisplatin, showed a strong synergistic effect, even when administered at low doses. Therefore, this combination regimen has the exciting potential to increase tumor-specific cytotoxicity and reduce drug-related side effects in patients with very little therapeutic options. Finally, due to the expression patterns of CD70, this study might also pave the way for improved treatment options in other tumor types.

## SUPPLEMENTARY MATERIALS FIGURES AND TABLES



## Abbreviations

ADCC: antibody-dependent cellular cytotoxicity
CDDP: cisplatin
CDDPi: CDDP+isotype control
DAPI: 4′,6-diamidino-2-fenylindool
E: effector cells
FBS: fetal bovine serum
GAPD: glyceraldehyde-3-phosphate dehydrogenase
IHC: Immunohistochemistry
MFI: mean fluorescence intensity
MICA A/B: major histocompatibility complex I chain-related molecule A and B
NK: Natural killer
NSCLC: Non-small cell lung cancer
PBMC: peripheral blood mononuclear cells
PBS: phosphate buffered saline
PE: phycoerythrin
PI: Propidium iodide
RPLA13: ribosomal protein L13a
RT-PCR: Real-time PCR
SDHA-1: Succinate dehydrogenase flavoprotein A1
SRB: sulforhodamine-B
T: target cells
TNF: tumor necrosis factor
ULBP2: UL16-binding protein 2
