# Supplementary material for: Preclinical data on the combination of cisplatin and anti-CD70 therapy in non-small cell lung cancer as an excellent match in the era of combination therapy
Source: Oncotarget. 2017 May 23;8(43):74058–67. doi: 10.18632/oncotarget.18202 (PMC5650323; doi:10.18632/oncotarget.18202)
Supplement: Supplementary file 1 [file oncotarget-08-74058-s001.pdf]

Preclinical data on the combination of cisplatin and anti-CD70 therapy in non-small cell lung cancer as an excellent match in the era of combination therapy

SUPPLEMENTARY MATERIALS

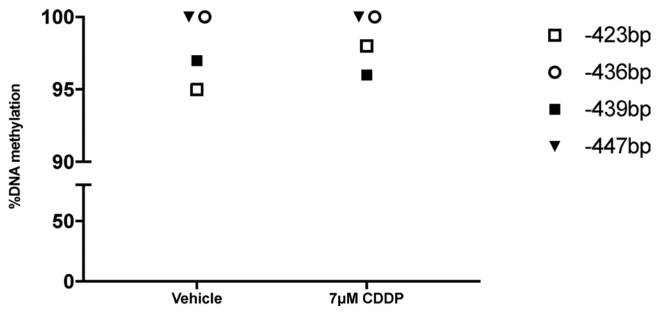

Supplementary Figure 1: No aberrations in DNA methylation of the CD70 promotor region post-treatment. Percentage DNA methylation of NCI-H1975 cells, 24 h after treatment with vehicle or CDDP (7 mM) at 4 different CpG sites in de the CD70 promotor region. Pyrosequencing was performed on a PyroMark Q24 instrument and cytosine methylation was quantified using the Pyromark Q24 software.

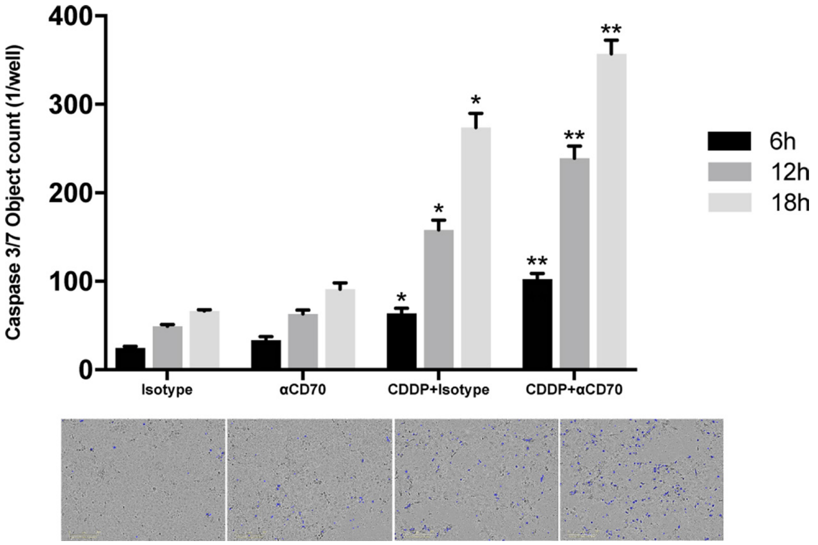

Supplementary Figure 2: Significant caspase-mediated cell death upon the combination of CDDP and αCD70. Top: Cells were treated with vehicle or CDDP (24 h, 7 µM). Cells were washed and aCD70 (0.5 µM) or isotype control (0.5 µM) was added to the medium in combination with NK cells (E/T=5/1) from healthy volunteers. Apoptosis was assessed 6 h, 12 h and 18 h after sequential treatment in 4 conditions: Isotype control, aCD70, CDDP + isotype control, CDDP + aCD70. Apoptotic cells, labelled by the IncuCyte™ Caspase 3/7 fluorescent reagent, are quantified in real time using the IncuCyte® ZOOM live-cell analysis system. Two replicates of the same condition were measured and run in parallel with NK cells from three different donors. Bars represent the mean ± SEM. Bottom: Representative IncuCyte™ images (10×) of NCI-H1975 cells, 12 h after treatment with Isotype control, aCD70, CDDP + isotype control and CDDP + aCD70. Apoptotic cells are indicated by a blue mask.

SUPPLEMENTARY MATERIALS AND METHODS

Methylation status of CD70 promotor region

Pyrosequencing

Cells were treated with vehicle or 7 μM CDDP for 24 h. DNA was isolated 24 h post-treatment using the 'GenElute™ Blood Genomic DNA Kit' (Qiagen, Venlo, The Netherlands). Bisulphite conversion was achieved using Epitect Fast DNA bisulphite kit (Qiagen), following the instructions of the manufacturer. Bisulphite-converted DNA was then used as a template for PCR. The primer sequences and PCR conditions are summarized in Supplementary Table 2. Amplification was carried out using the PyroMark PCR kit (Qiagen), following the instructions of the manufacturer. Next, biotin-labeled PCR products were captured with Streptavidin Sepharose beads (GE healthcare, UK) and made single stranded using a pyrosequencing vacuum prep Tool (Qiagen). Pyrosequencing was performed on a PyroMark Q24 instrument (Qiagen) and cytosine methylation was quantified using the Pyromark Q24 software.

Cytotoxicity assays

IncuCyte™

Real-time measurement of ADCC was performed using the incuCyte™ (Essen Bioscience, Hertfordshire, United Kingdom). NCI-H1975 cells were seeded at 5×10^3 cells/well, incubated overnight and exposed to 7 μM CDDP (IC20-value A549 cell line) or vehicle for 24 h. One day after treatment, medium was aspirated and replaced with medium containing the IncuCyte™ Caspase-3/7 Apoptosis Reagent (Essen Bioscience). Cells were treated with ARGX-110 (arGEN-x BVBA, Zwijnaarde, Gent, 0.5 μM) or isotype control (I5029, Sigma-Aldrich, Diegem, Belgium, 0.5μM) in combination with effector (E) NK cells (E:T=5:1) and cell-death was assessed for 18 h.

Supplementary Table 1: Cellular characteristics of NSCLC cell lines and IC50-values of CDDP monotherapy

Cell line	Histological subtype	Genetic aberrations	IC_{50} CDDP (μM)
LUDLU-1	Squamous	P53 mutation	5.46 ± 0.93
NCI-H1650	Adenocarcinoma	P53 intron mutation Activating EGFR mutation	6.51 ± 0.46
NCI-H1975	Adenocarcinoma	P53 mutation Inactivating EGFR mutation	19.34 ± 1.72
HCC827	Adenocarcinoma	Activating EGFR mutation	15.95 ± 1.37
A549	Adenocarcinoma	KRAS mutation	10.04 ± 0.72

Data on genetic aberrations retrieved from Cell Lines Project (COSMIC)

Supplementary Table 2: CpG pyrosequencing primers

Gene	PCR primers[a]	Temp	Sequencing primer[a]
**CD70**	FW:5′-TGTGTTGTGTATTGGGGATATAG-3′<br>REV:5′-Biotin-ACATAATAAAACCCCATCTCTACTAA-3′	59°C	TTAGGTTGGAGTGTAG

[a]Primers were designed using the Pyromark Assay Design v2.0 software (Qiagen).
